# Studies on the Vitrified and Cryomilled Bosentan

**DOI:** 10.1021/acs.molpharmaceut.1c00613

**Published:** 2021-12-01

**Authors:** Aldona Minecka, Krzysztof Chmiel, Karolina Jurkiewicz, Barbara Hachuła, Rafał Łunio, Daniel Żakowiecki, Kinga Hyla, Bartłomiej Milanowski, Kajetan Koperwas, Kamil Kamiński, Marian Paluch, Ewa Kamińska

**Affiliations:** † Department of Pharmacognosy and Phytochemistry, Faculty of Pharmaceutical Sciences in Sosnowiec, Medical University of Silesia in Katowice, ul. Jagiellonska 4, 41-200 Sosnowiec, Poland; ‡ Institute of Physics, Faculty of Science and Technology, University of Silesia in Katowice, 75 Pułku Piechoty 1, 41-500 Chorzów, Poland; § Institute of Chemistry, University of Silesia in Katowice, 40-006 Katowice, Poland; ∥ Polpharma SA, 83-200 Starogard Gdański, Poland; ⊥ Chemische Fabrik Budenheim KG, Rheinstrasse 27, 55257 Budenheim, Germany; # Chair and Department of Pharmaceutical Technology, Faculty of Pharmacy, Poznan University of Medical Sciences, 60-780 Poznan, Poland; ∇ GENERICA Pharmaceutical Lab, Regionalne Centrum Zdrowia Sp. z o.o., Na Kępie 3, 64-360 Zbąszyń, Poland

**Keywords:** bosentan, vitrification, cryomilling, molecular mobility, water removal, dissolution rate

## Abstract

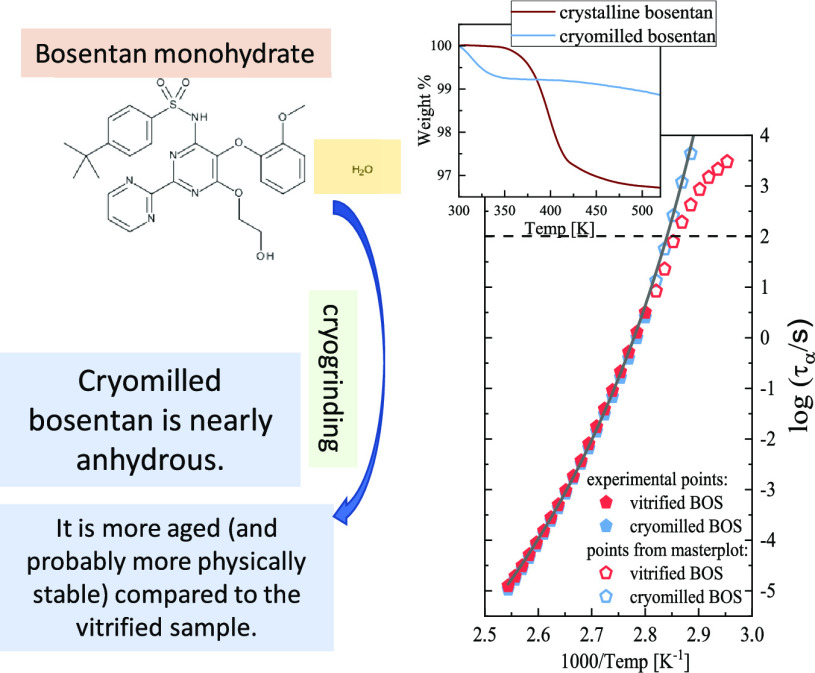

In this paper, several experimental techniques [X-ray diffraction, differential scanning calorimetry (DSC), thermogravimetry, Fourier transform infrared spectroscopy, and broad-band dielectric spectroscopy] have been applied to characterize the structural and thermal properties, H-bonding pattern, and molecular dynamics of amorphous bosentan (BOS) obtained by vitrification and cryomilling of the monohydrate crystalline form of this drug. Samples prepared by these two methods were found to be similar with regard to their internal structure, H-bonding scheme, and structural (α) dynamics in the supercooled liquid state. However, based on the analysis of α-relaxation times (dielectric measurements) predicted for temperatures below the glass-transition temperature (*T*_g_), as well as DSC thermograms, it was concluded that the cryoground sample is more aged (and probably more physically stable) compared to the vitrified one. Interestingly, such differences in physical properties turned out to be reflected in the lower intrinsic dissolution rate of BOS obtained by cryomilling (in the first 15 min of dissolution test) in comparison to the vitrified drug. Furthermore, we showed that cryogrinding of the crystalline BOS monohydrate leads to the formation of a nearly anhydrous amorphous sample. This finding, different from that reported by Megarry et al. [Carbohydr. Res.2011, 346, 1061−10642149283010.1016/j.carres.2011.03.011] for trehalose (TRE), was revealed on the basis of infrared and thermal measurements. Finally, two various hypotheses explaining water removal upon cryomilling have been discussed in the manuscript.

## Introduction

Over the years, the pharmaceutical industry developed a number of approaches that led to the increase of the solubility, dissolution rate, and ultimately bioavailability of poorly water-soluble drugs from solid dosage forms. It turned out that the most common and efficient way to enhance these important properties is the chemical transformation of the active pharmaceutical ingredient (API) into its salt.^1^ Unfortunately, only about 20–30% of the new potential API molecules may undergo such chemical conversion. Therefore, other methods are being sought to modify the solubility of the remaining 70–80% of pharmaceuticals.^2^ Herein, one can list the most popular ones, including the formation of solid lipid nanoparticles,^3^ liposomes,^4^ micelles,^5^ soft gelatine capsules,^6^ co-crystals,^7^ or metastable polymorphs.^8^ Simultaneously, in recent years, a change of the physical state of the sample, i.e., amorphization, has attracted increasing attention of scientists working in this field.^9−13^

In general, amorphous APIs can be obtained via methods based on mechanical activation of a crystalline mass (e.g., during milling/grinding),^14−18^ solvent removal (e.g., freeze- or spray-drying),^10,19−23^ and temperature variation (e.g., vitrification).^24,25^ Each of them has its advantages and disadvantages. Regarding the temperature-based methods, the amorphous form of the desired active substance is made by the fast cooling of the melt. In recent years, this approach has been gaining increasing interest, especially considering the extrusion-based production techniques (e.g., hot melt extrusion). However, it has a certain limitation. Namely, it is not suitable for compounds undergoing thermal degradation at the melting point.^26,27^ There are also systems very sensitive to solvents.^28,29^ In such cases, methods involving mechanical activation of crystalline material using various types of mills seem to be a quite interesting alternative. Intensive studies on this topic by Descamps’ group revealed that the balance between the temperature of milling and the glass-transition temperature (*T*_g_) of API plays a crucial role in the effectiveness of amorphization.^30−32^ They have shown that when the process is carried out at temperatures (*T*) significantly below *T*_g_ (e.g., grinding in a liquid nitrogen atmosphere—cryomilling), amorphization is promoted. If *T* are close or above the *T*_g_ of the sample (e.g., room temperature), the material undergoes polymorphic transformations.^33,34^ More importantly, in some cases, milling allows for the production of metastable polymorphs not achievable by other methods.^27,33,35,36^ However, even this preparation technique has its limitations. Namely, the physical stability of the amorphous substance obtained via grinding of the crystalline solid might differ significantly from that produced by rapid cooling of a molten sample. Such a situation occurred in indomethacin, where the amorphous substance prepared by grinding recrystallized over 1000 times faster than the one obtained by vitrification.^27,37^ Moreover, even if milling is one of the most effective ways to lower the activation barrier for different types of chemical reactions (tribochemistry),^38^ many substances undergo decomposition during this process.^39^ To avoid this undesired effect, very often, the temperature of the milling is lowered (cryomilling/cryogrinding).^40^ However, it should be pointed out that there is a reported case of the API—furosemide—which undergoes chemical decomposition during cryogenic grinding alone as well as in the system with inulin or poly(vinylpyrrolidone) (PVP) polymer.^41^ It was clearly shown that the character of the excipient has a tremendous impact on the progress of chemical decomposition of this popular diuretic. Herein, one can stress that the presence of substances forming hydrogen bonds (e.g., inulin or PVP) catalyzed this process, while those characterized by van der Waals interactions (acetylated saccharides) stabilized API (inhibited its dissociation).^42^ Therefore, a better understanding of the nature of the amorphous materials obtained by milling seems to be of fundamental importance.

Based on the reported data, one can distinguish several main ideas explaining the crystalline-to-amorphous transformation upon mechanical grinding.^43−47^ The most popular concept proposed by Martin and Bellon explains the mutual dependence between the temperature of grinding and the effectiveness of amorphization.^44^ The authors postulated that the crystalline-to-amorphous transition is a result of two competing processes: a disordering process induced by milling (thermally independent) and a recovery process (thermally dependent). Another approach suggests that amorphization might be understood as a milling-induced disorder generated by a large number of mechanical perturbations (reduction of crystallite size, accumulation of crystallite defects, polymorphic transformations, and partial or complete amorphization).^46,47^ Researchers indicate that during grinding, the progressive defects in the crystalline lattice increase the overall energy of the system, which, in turn, constitutes the necessary thermodynamic-driven force to initiate the transformation.^43^ Finally, it is also worth mentioning the concept considering the local heating effect. It assumes that the release of energy during a mechanical impact leads to the local heating of the sample, which is instantaneously cooled very fast. Herein, it should be noted that intensive studies by Descamps’ group on the progress of mutarotation in milled lactose and glucose indicated that this idea is not reliable.^35,48^

In this paper, we present the comparative analysis of the amorphous bosentan (BOS) obtained by both vitrification and cryomilling of the crystalline monohydrate form of this API. Bosentan (BOS) is an endothelin-1 receptor antagonist dedicated for the treatment of pulmonary arterial hypertension (PAH) in adults. It decreases both systemic vascular resistance and pulmonary vascular resistance, and consequently increases cardiac output without increasing the heart rate.^16,49^ Due to the fact that BOS is poorly soluble in water and a highly permeable compound, it is classified as a class IIa drug within the Biopharmaceutical Classification System (BCS). Amorphization, therefore, seems like a sound step in the enhancement of its solubility/dissolution rate. Herein, we confirmed that one is able to obtain BOS with a negligible water content as a result of cryomilling. Furthermore, we found out that regardless of the chosen method of amorphization (vitrification or cryogrinding), the samples are nearly identical in terms of the local structure and H-bonding scheme. However, they differ significantly with respect to their dynamical properties below the *T*_g_, as well as in dissolution rates with respect to the crystalline form of API.

## Experimental Section

### Materials and Methods

#### Material

**Scheme 1:**
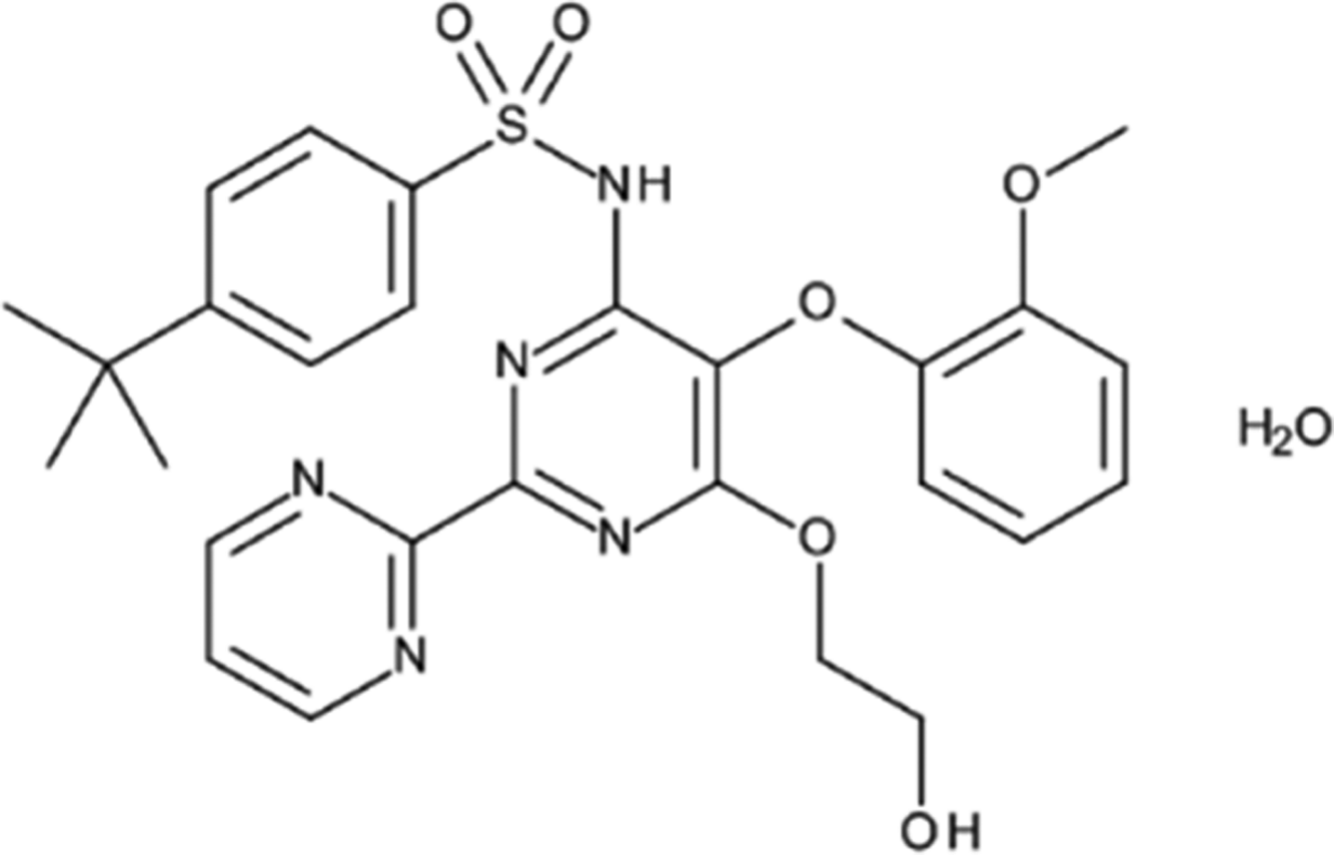
Chemical Structure of BOS Monohydrate

Bosentan monohydrate of molecular weight *M*_W_ = 569.6 g mol^–1^ and purity ≥ 99% was donated by Polpharma (Starogard Gdański, Poland) and used without further purification. Its chemical structure is presented in Scheme 1.

#### Preparation of Amorphous Samples

Preparation of the vitrified system in the case of calorimetric measurements was done by melting of the crystalline bosentan monohydrate at *T* = 500 K—during the first differential scanning calorimetry (DSC) scan—and vitrification by fast cooling (20 K min^–1^) during the second DSC scan. Sample preparation for the X-ray diffraction (XRD), Fourier transform infrared spectroscopy (FTIR), and broad-band dielectric spectroscopy (BDS) studies involved melting at *T* = 500 K, followed by vitrification on a previously chilled copper plate. All measurements were performed immediately after preparation of the amorphous systems to avoid recrystallization.

Grinding was carried out using a cryogenic impact mill (CryoMill ∼100–240 V-50/60 Hz, Retsch GmbH, Germany) consisting of a stainless steel vessel immersed in liquid nitrogen, within which a stainless steel rod is vibrated by means of a magnetic coil. Before grinding, a 5 min precool time was programmed. Then, the mill was planned to have an impact frequency of 9 cycles per second for 12 min grinding periods separated by 3 min cool–down periods. The total milling time for our sample was 2.5 h. After this time, the grinding vial was opened and the sample was immediately transferred for thermogravimetric analysis (TGA) and DSC investigations. The freshly prepared cryomilled BOS was a yellow powder.

#### X-ray Diffraction (XRD)

XRD measurements were performed on a Rigaku-Denki D/MAX RAPID II-R diffractometer equipped with an image plate detector, a rotating Ag anode, and an incident beam (002) graphite monochromator (wavelength of the incident beam λ = 0.56 Å). Samples were measured in borosilicate glass capillaries, in the Debye–Scherrer geometry. The diffraction data were collected as two-dimensional patterns and converted into one-dimensional functions of scattering intensity versus scattering angle 2θ. Finally, the background intensity from the empty capillary was subtracted.

#### Differential Scanning Calorimetry (DSC)

The thermodynamic properties of BOS samples were examined using a Mettler Toledo DSC 1 STARe System (Columbus, OH). The measuring device was equipped with an HSS8 ceramic sensor having 120 thermocouples. The instrument was calibrated for temperature and enthalpy using indium and zinc standards. The melting points were determined as the onset of the peak, whereas the glass-transition temperatures were determined as the midpoint of the heat capacity increment. The samples were placed in aluminum crucibles (40 μL). All measurements were carried out in the range of 300–500 K with a 10 K min^–1^ heating rate.

#### Thermogravimetric Analysis (TGA)

Water evaporation from BOS (crystalline and cryomilled samples) was examined by a Mettler TG 50 thermogravimetric analyzer linked to a Mettler MT5 balance (Mettler Toledo, Switzerland). The powder in open aluminum pans was placed in a furnace under nitrogen purge (30 mL min^–1^) and heated at 10 K min^–1^ from room temperature to *T* = 1150 K. Water evaporation of the sample was determined by the weight loss percentage.

#### Fourier Transform Infrared (FTIR) Spectroscopy

FTIR spectra of the crystalline BOS monohydrate, as well as vitrified and cryomilled API, were collected in attenuated total reflectance (ATR) mode using a Thermo Scientific Nicolet iS50 spectrometer equipped with a built-in ATR accessory. The samples were placed on the ATR crystal and measured at a resolution of 4 cm^–1^ in the wavenumber range of 400–4000 cm^–1^. Baseline corrections were performed using OMNIC software.

#### Broad-Band Dielectric Spectroscopy (BDS)

Dielectric measurements of the amorphous (vitrified and cryomilled) BOS were carried out using Novo-Control GMBH Alpha dielectric spectrometer (Montabaur, Germany), in the frequency range of 10^–1^–10^6^ Hz, at given temperatures, with a heating rate equal to 0.5 K min^–1^. The temperature was controlled by a Quatro temperature controller with temperature stability better than 0.1 K. The samples were placed in a parallel-plate cell made of stainless steel (diameter 15 mm, and 0.1 mm gap with quartz spacer).

#### Intrinsic Dissolution Testing

The intrinsic dissolution rate (IDR) of the crystalline, cryomilled, and vitrified BOS samples was measured by the stationary disc system^50^ using an Erweka DT60 paddle dissolution testing apparatus (ERWEKA GmbH, Germany). About 100 mg of API was compressed at 1500 psi and held for 60 s in a die to form compacted disks of 8 nm diameters and the same thickness (to eliminate the effect of different specific areas of both materials). Dissolution studies were performed in 500 mL of 1% w/v sodium dodecyl sulfate (SDS) in ultrapure water^51^ maintained at 37 ± 0.5 °C and a paddle rotation speed of 100 rpm. They were carried out for 120 min, and the concentration of API was determined on-line every 5 min by a UV–vis spectrophotometer Nicolet Evolution 300 (Thermo Electron Corporation) at 264 nm (signal) and 450 nm (background correction). All measurements were performed in triplicate. IDR, the rate of mass transfer from solid to liquid state, when conditions such as surface area, pH, ionic strength, and stirring speed are kept constant, was determined using the following equation:^52^
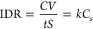
1where *C* is the drug concentration at time *t*, *V* is the volume of the test solution, *S* is the disk’s surface area, *k* is the intrinsic dissolution rate constant, and *C*_s_ is the saturation solubility of the drug. The IDRs were calculated from each curve’s slope for periods of 0–120 min for crystalline BOS, and 0–15 and 60–120 min for vitrified and cryomilled BOS, respectively.

## Results and Discussion

**Figure 1:**
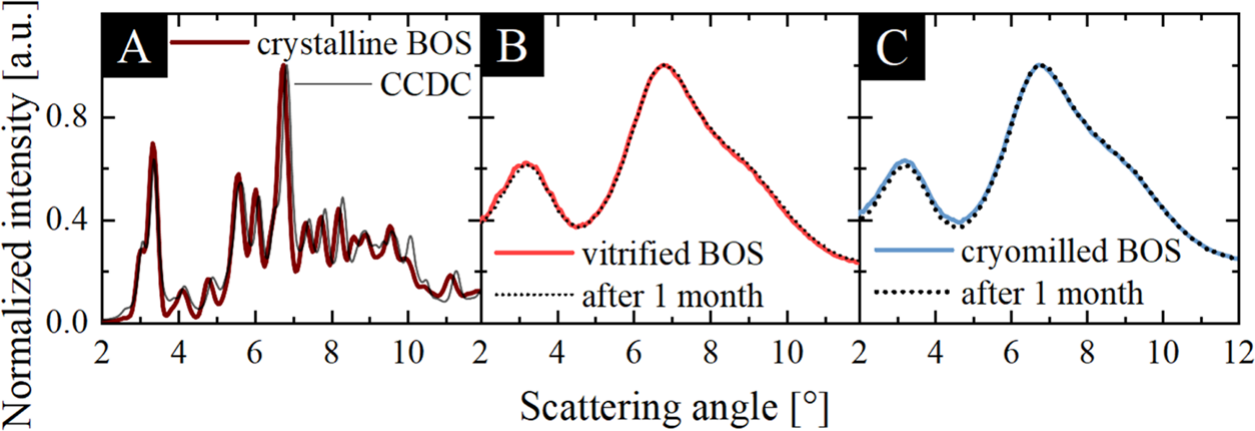
X-ray diffraction patterns of (A) crystalline BOS monohydrate and its reference from CCDC database, (B) vitrified BOS just after preparation and after 1 month of storage, and (C) cryomilled BOS just after preparation and after 1 month of storage.

At the beginning of the study, we prepared amorphous BOS systems employing two methods: cryomilling and vitrification. To determine whether the samples were fully amorphous after processing, XRD measurements were carried out. In Figure 1A, XRD patterns of the studied crystalline BOS monohydrate and its reference from the Cambridge Crystallographic Data Centre (CCDC, no. 920210) are overlapped (wine and black lines, respectively). The XRD data in a wider 2θ range are presented in the Supporting Information (Figure S1). The agreement between both XRD patterns confirms that the initial crystalline BOS has a monohydrate form. According to literature data, this BOS monohydrate crystallizes in the monoclinic *P*2_1_/*c* system (unit cell parameters: 12.3393(4), 15.1238(6), 14.6988(4) Å, unit cell angles: 90.0, 95.037(3), 90.00°) with four molecules per unit cell.^53^Figure 1B,C shows XRD data for the amorphous BOS produced by vitrification and cryomilling (light red and blue lines, respectively). It can be seen that the XRD patterns are typical for the disordered structures with no long-range crystalline order. That means both methods were successfully used to amorphization of BOS monohydrate. However, one can see that the shape of the XRD patterns for disordered API resembles the broadened pattern of the crystalline sample, indicating that the main structural motif of the local structure of the crystalline form may be preserved in the amorphous phase. The pre-peak appearing in the XRD patterns of the disordered samples around 3.2° before the main diffraction peak is the fingerprint of the supramolecular structure. It signifies the presence of supramolecular assemblies and the medium-range order in the amorphous BOS. However, since the applied vitrification and cryomilling caused a transformation of the molecular structure from crystalline toward more disordered, we will refer to them as “amorphous” samples.

A comparison of the diffractograms for the vitrified and cryomilled API (Figure 1B,C) exhibits that they are very similar. The diffraction data normalized to unity are characterized by almost identical peak positions, widths, and intensities. Moreover, the atomic pair distribution functions of the studied materials, shown in Figure S2 in the Supporting Information, are almost identical at both short- and medium-range scales. Hence, one can state that the atomic-scale structures of both samples are very similar. Moreover, after 1 month of storage at room temperature (relative humidity ranging between 40 and 50%) and under ambient pressure, the diffraction patterns of vitrified and cryomilled BOS did not show any changes compared to those collected immediately after preparation (see the solid and dotted lines in Figure 1B,C).

The described similarities between the amorphous BOS samples, especially the ones indicating the presence of supramolecular structures, raise the question whether there is water in the cryoground sample that should have some impact on the spatial rearrangement of the molecules. To test this hypothesis and characterize the amorphous BOS samples prepared via vitrification and cryomilling in more detail, we performed studies with the use of DSC, thermogravimetry, and FTIR methods.

**Figure 2:**
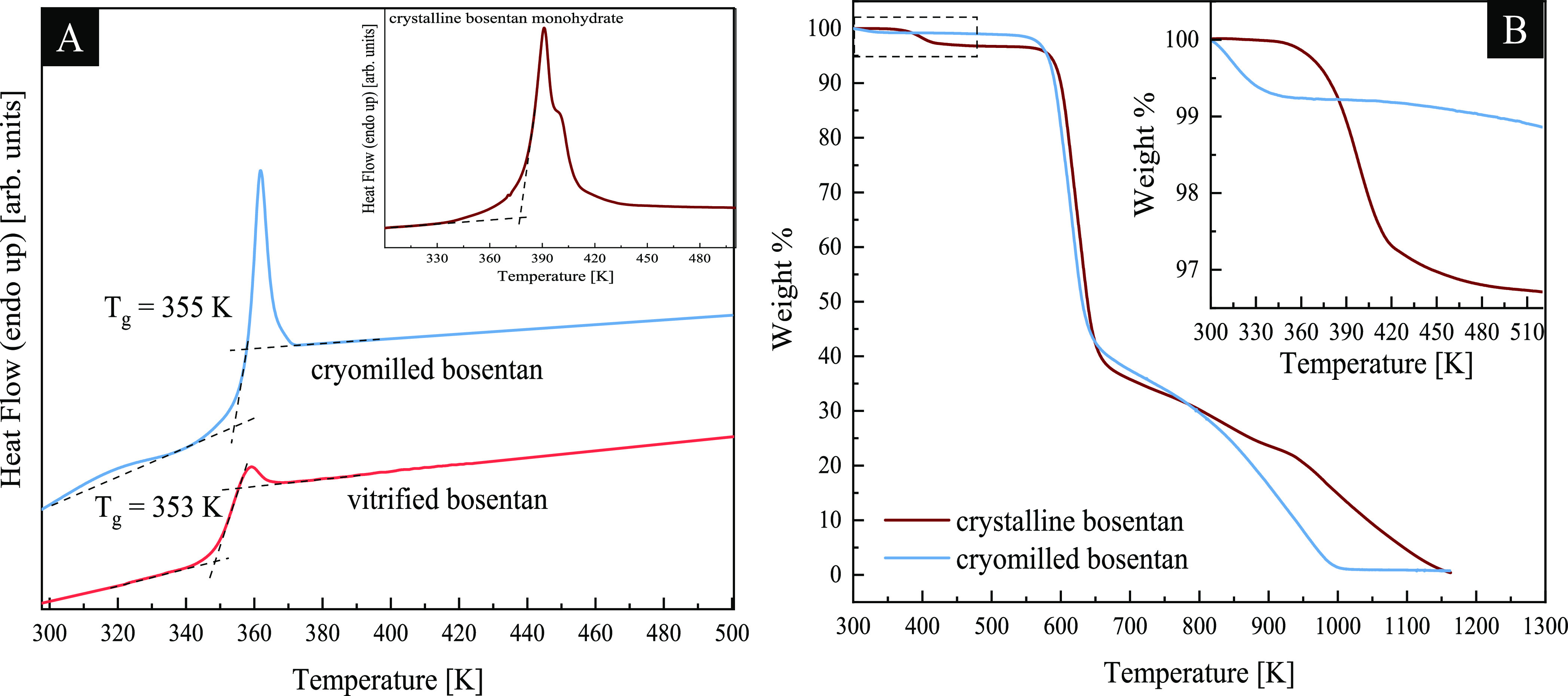
(A) DSC thermograms of cryomilled and vitrified BOS and (inset) crystalline BOS monohydrate. (B) TGA trace of crystalline and cryomilled BOS samples.

First, the thermal properties of crystalline bosentan (BOS) monohydrate, as well as two amorphous samples, were determined. The thermograms obtained from DSC measurements carried out in the temperature range of 300–500 K with a heating rate of 10 K min^–1^ are presented in Figure 2A. It can be seen that the crystalline sample (wine line in the inset) exhibits an endothermal peak, which is not singular—there is an additional trailing edge to it. Such behavior results from the overlapping of two processes: the melting of the sample with the onset at 379 K and the evaporation of the hydration water. On the other hand, DSC curves of the vitrified (light red line) as well as cryomilled (light blue line) substances are characterized by the presence of a single step-like thermal event that corresponds to the glass transition. The exact values of the *T*_g_’s are similar and equal to 353 and 355 K for the BOS samples obtained by melt quenching and cryomilling, respectively. Importantly, the increase in the enthalpy recovery is much stronger for the cryomilled sample in comparison to the vitrified one. According to the literature data, such a great overshoot in enthalpy in the former sample indicates that it exhibits the characteristics of material aged for a long time. A similar scenario was observed for amber glass aged for billion years.^54^ Hence, one can state that the amorphous API obtained by cryomilling is more aged with respect to the vitrified one. Moreover, both amorphous samples did not show any tendency toward recrystallization during the nonisothermal calorimetric studies. Furthermore, the absence of melting peaks can be taken as further evidence that both methods of preparation (vitrification and cryomilling) ensure fully amorphous BOS.

**Figure 3:**
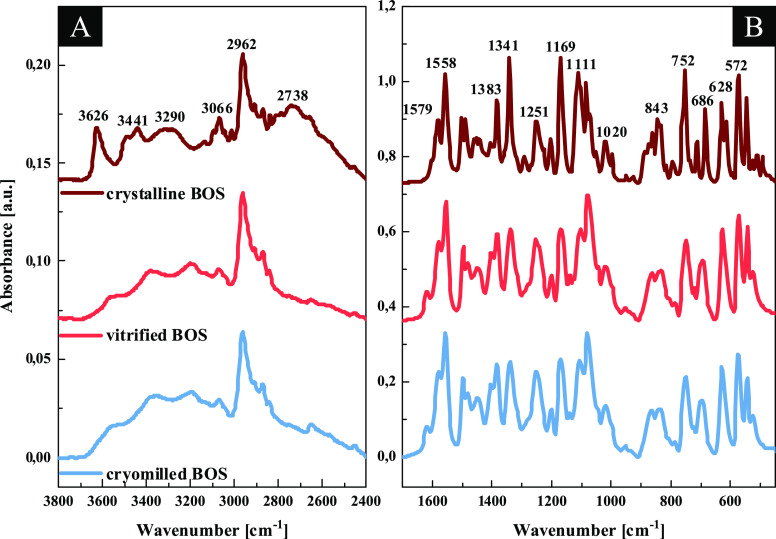
FTIR spectra of the crystalline BOS monohydrate (wine line), amorphous BOS produced via vitrification (light red line), and cryomilling (light blue line). The data are presented in two spectral regions: (left) 3800–2400 cm^–1^ and (right) 1700–450 cm^–1^. The spectra were normalized with respect to the absorbance maximum in the selected frequency ranges and plotted with an offset for clarity.

As complementary to DSC, thermogravimetric measurements were carried out on the crystalline and cryoground BOS. TGA curves of both samples are presented in Figure 2B (solid wine and light blue line, respectively). During this experiment, two weight loss steps were observed in each case: the first, a loss of almost 3.3 wt % (crystalline monohydrate) or 0.7 wt %—endothermal peak near 300–320 K (cryoground substance), which can be identified as water evaporation, and the second with an onset at approximately 600 K, corresponding to thermal decomposition of the sample. Note that the water removal during melting of the crystalline BOS monohydrate is in good agreement with the literature.^55^ Importantly, a weight loss of approximately 0.7 wt % indicates a strongly reduced water content compared to the crystalline BOS (weight loss 3.3 wt %). It means that its large amount has been removed from the cryomilled sample (note that the residual water molecules are located between API molecules). To confirm this experimental observation as well as to check whether the H-bonding scheme is the same in both amorphous BOS systems, we performed further FTIR investigations. The infrared spectrum of the crystalline BOS, commercially available in the form of monohydrate (BOS·H_2_O), was also measured for comparison. Figure 3A,B illustrates the representative FTIR spectra of these three samples obtained in the two frequency ranges, 3800–2400 cm^–1^ and 1700–450 cm^–1^, respectively. Moreover, the detailed FTIR bands’ assignments of crystalline BOS·H_2_O, correlated with DFT calculations, are presented in the Supporting Information. It is clearly visible that FTIR spectra of amorphous BOS prepared by the two methods (light blue and light red lines in Figure 3A,B) resemble that of the crystalline sample, especially in the lower-frequency range (below 1700 cm^–1^). However, both samples have broader peaks than the crystalline one, and the peak splitting partially disappears in these systems. It means the destruction of long-range crystalline order after vitrification as well as cryomilling of BOS monohydrate. The noticeable spectral differences between the analyzed spectra can be found in the regions associated with the formation of H-bonds, i.e., 3700–2400 and 1620–1520 cm^–1^. In the disordered BOS, the X–H band contour (3550–3200 cm^–1^) changes and the peak at 3626 cm^–1^ (assigned to the vibrations of water in the collected spectra) disappears. Moreover, the shoulder occurring between 3000 and 2400 cm^–1^ is not detected for amorphous samples. These experimental results are evidence of a reorganization of the H-bond network of API and the resulting variations in the geometrical parameters of the X–H bonds, as the water content is strongly reduced. In this place, one can notice that the spectral profiles of amorphous BOS obtained via two various methods are nearly the same. It indicates a similar H-bonding scheme in these systems and much different with respect to the crystalline API.

Hence, FTIR data are consistent with the outcome of the TGA investigations that revealed a significantly lower amount of water in the cryoground amorphous BOS with respect to the crystalline BOS monohydrate.

Importantly, this striking and intriguing result is different from that reported by Megarry et al. for trehalose (TRE), which is however not a hydrophobic, but a hydrophilic sample.^56^ In the recalled work, the authors postulated that cryogenic milling is the only method to obtain hydrated amorphous substances. Just to mention that due to cryomilling of the crystalline trehalose (TRE) dihydrate, they always obtained a hydrated disordered sample. Therefore, a few important questions arise. Why the result described by us and the one reported by Megarry et al. are so different? From the analysis of the chemical structure of BOS and TRE as well as their solubility in water, we can easily find that the former system is more hydrophobic and almost insoluble in this solvent, while TRE has plenty of hydroxyl units capable of forming strong intermolecular HBs in aqueous solutions (consequently, it dissolves in water very well). In fact, one can postulate that the hydrophobic character of BOS underlies water removal from the sample. However, in addition, there must be other factors responsible for the water disappearance/evaporation. Based on the diffractograms and FTIR spectra indicating the same local structure and H-bonding interaction pattern of the cryomilled and vitrified BOS, one can speculate that upon cryogrinding, local heating of the sample around *T* ∼ 393 K occurs. This process is followed by instantaneous quenching. However, if this hypothesis is true, the water removal should be detected also in the case of cryoground trehalose dihydrate since the melting temperature of hydrated saccharide is comparable to that of BOS monohydrate. Therefore, it seems that this explanation does not hold anymore. Hence, to explain the differences between our data and those reported earlier by Megarry et al.,^56^ one can take into account the hermeticity of the vessel. In the case of our investigations, a vial that is hermetically closed at *T* = 298 K and then cooled down to *T* ∼ 80 K was used. Such a dramatic decrease in temperature is surely accompanied by a drop in pressure inside the vessel. However, it is not as significant as in the case of the ordinary freeze-drying process. Next, due to water release from the crystalline lattice during milling, it solidifies as ice, and then it is removed via sublimation, just as it happens during lyophilization. Importantly, this effect most likely can be observed only in the mills, where the vessel is hermetically closed (RETSCH Mill). In other cases, such a phenomenon may not be detected. However, it is just a hypothesis that must be verified experimentally in the future by investigating other hydrates in different kinds of grinders. It is worth to stress that at the moment, we have no other reasonable explanation on the water removal upon cryogrinding of the examined API.

**Figure 4:**
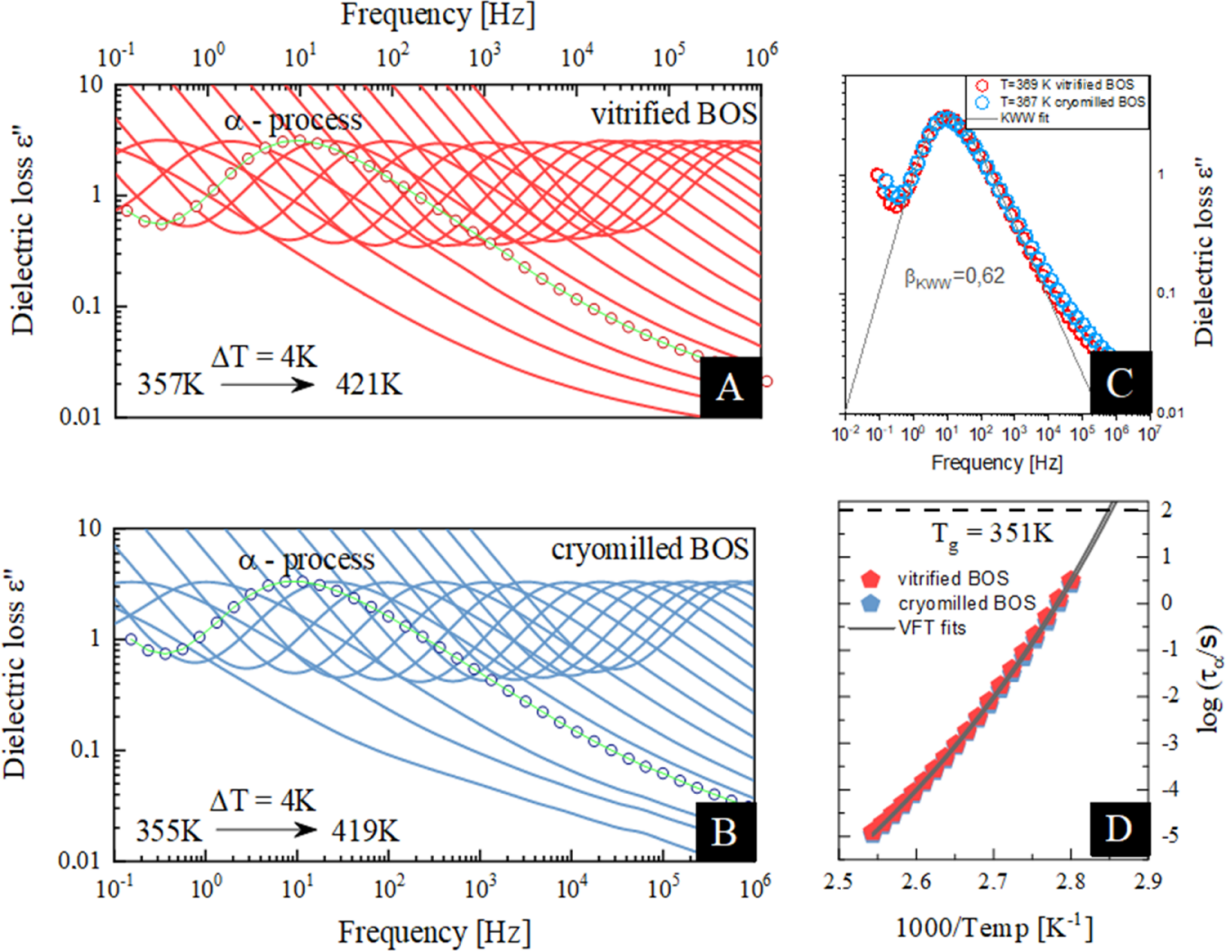
(A, B) Representative dielectric loss spectra of amorphous BOS above its glass-transition temperature. Light red lines correspond to the vitrified sample, while light blue lines correspond to the cryomilled one. Solid green lines through the points (open circles) are HN fits to the spectra measured at 369 and 367 K, respectively. (C) Comparison of the shape of α-process in the vicinity of *T*_g_ (for the same τ_α_). The black line represents KWW fit. (D) Relaxation map of BOS. Red and blue pentagons correspond to the vitrified and cryomilled samples. The temperature dependence of structural relaxation times, τ_α_, in the supercooled liquid state has been described by the VFT equation (solid gray lines).

As the following step, we decided to apply dielectric spectroscopy to assess the molecular mobility of amorphous BOS prepared via different techniques. Taking into consideration the results provided from thermogravimetric and infrared studies, that both amorphization methods result in nearly/fully anhydrous BOS, one can exclude the impact of water on the molecular dynamics both above and below samples’ *T*_g_s. During the heating of the vitrified and cryomilled API (the direction of BDS experiments was from low *T* to high *T*), one can observe a well-resolved peak in dielectric spectra corresponding to the structural (α) relaxation process; see Figure 4A,B. This peak is well visible at temperatures above 357 K (i.e., in the supercooled liquid region) and moves toward higher frequencies with increasing temperature. We fitted the selected α-loss peak for each sample (*f*_max_ ∼ 10 Hz) by means of the one-sided Fourier transform of the Kohlrausch–Williams–Watts (KWW) function to characterize its shape;^57,58^ see Figure 4C (black line). It should be emphasized that the value of the β_KWW_ parameter (*T* ∼ *T*_g_) is the same, equal to 0.62, regardless of the amorphization method. Furthermore, we found that the shape of the α-loss peak is nearly insensitive to temperature change (see Figure S5 in the Supporting Information). It is worth mentioning that β_KWW_ might vary from 0 to 1. If this parameter is approaching 0, the shape of the structural relaxation is asymmetric and broad. However, if the value of β_KWW_ is equal to 1, it means that the α-relaxation peak is symmetric and narrow, which corresponds to the Debye case. BOS having β_KWW_ = 0.62 and Δε_α_ = 7.8, similarly to the case of celecoxib (β_KWW_ = 0.67, Δε_α_ = 8.3),^59^ eugenol (β_KWW_ = 0.68, Δε_α_ = 8.0),^60^ and 2-phenyl-5-acetomethyl-5-ethyl-1,3-dioxocyclohexane (β_KWW_ = 0.64, Δε_α_ = 8.0),^61^ follows the anticorrelation between the narrowness of α-loss peak (high value of β_KWW_) and its dielectric strength (Δε_α_) in the van der Waals glass formers.^62^ Note that the Δε_α_ parameter for the examined API was determined from the analysis of loss spectra recorded above the *T*_g_ using the Havriliak–Negami (HN) function with the conductivity term (eq 2)^63^
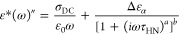
2where ε_0_ is the permittivity of vacuum, σ_DC_ is the direct current (dc) conductivity, ω is equal to 2π*f* (where *f* is the frequency of the maximum of the peak), τ_HN_ is the HN relaxation time, *a* and *b* are the parameters that represent the symmetric and asymmetric broadening of the relaxation peak, respectively (representative fits of the HN function to the dielectric spectra are presented in Figure 4A,B—green lines). From the mentioned fitting procedure, we also determined the dependencies of α-relaxation times (τ_α_) in a broad temperature range for the vitrified and cryomilled API systems. It should be added that τ_α_, recalculated from τ_HN_ according to the following formula (eq 3)^63^
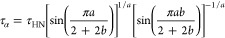
3is presented in Figure 4D as light red and blue pentagons. The temperature evolution of structural relaxation times usually shows non-Arrhenius-like behavior in the supercooled liquid region. Therefore, to parameterize it, we applied the Vogel–Fulcher–Tammann (VFT) equation, which is defined as follows^64−66^
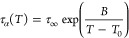
4where τ_∞_, *B*, and *T*_0_ are fitting parameters. To determine the values of *T*_g_, a well-known definition, where *T*_g_ = *T* (τ_α_ = 100 s) was used. Accordingly, from the extrapolation of VFT fit to 100 s, we estimated *T*_g_ of both vitrified and cryomilled samples as 351 K. Note that the obtained value was close to those determined from DSC studies (353 and 355 K, respectively) considering the different heating rates applied during the measurements.^67^ Furthermore, both presented τ_α_(*T*) dependencies are in very good agreement, which implies that regardless of the preparation method, amorphous BOS exhibits the same structural dynamics in the supercooled liquid state.

As reported in the case of indomethacin, the physical stability of the amorphous material obtained via milling of the crystalline solid differs significantly from that prepared through a rapid cooling of a molten sample. In the recalled case, the cryomilled material recrystallized over 1000 times faster than the one obtained by vitrification.^27,37^ Even though throughout the dielectric measurements, neither of the samples exhibited any tendency toward recrystallization, we made an attempt to determine the long-term physical stability of the samples.

In recent years, a number of reports suggested that the recrystallization of the sample at temperatures below its *T*_g_ depends on the sample’s global mobility. It has been demonstrated on the examples of several amorphous APIs, such as bicalutamide,^68^ ezetimibe,^69^ and griseofulvin,^70^ that the recrystallization’s timescale is of the same order of magnitude as the value of τ_α_ in the glassy state. Therefore, one might be able to predict the approximate long-term physical stability of a given amorphous API, based only on the τ_α_ predicted for temperatures below its *T*_g_.

**Figure 5:**
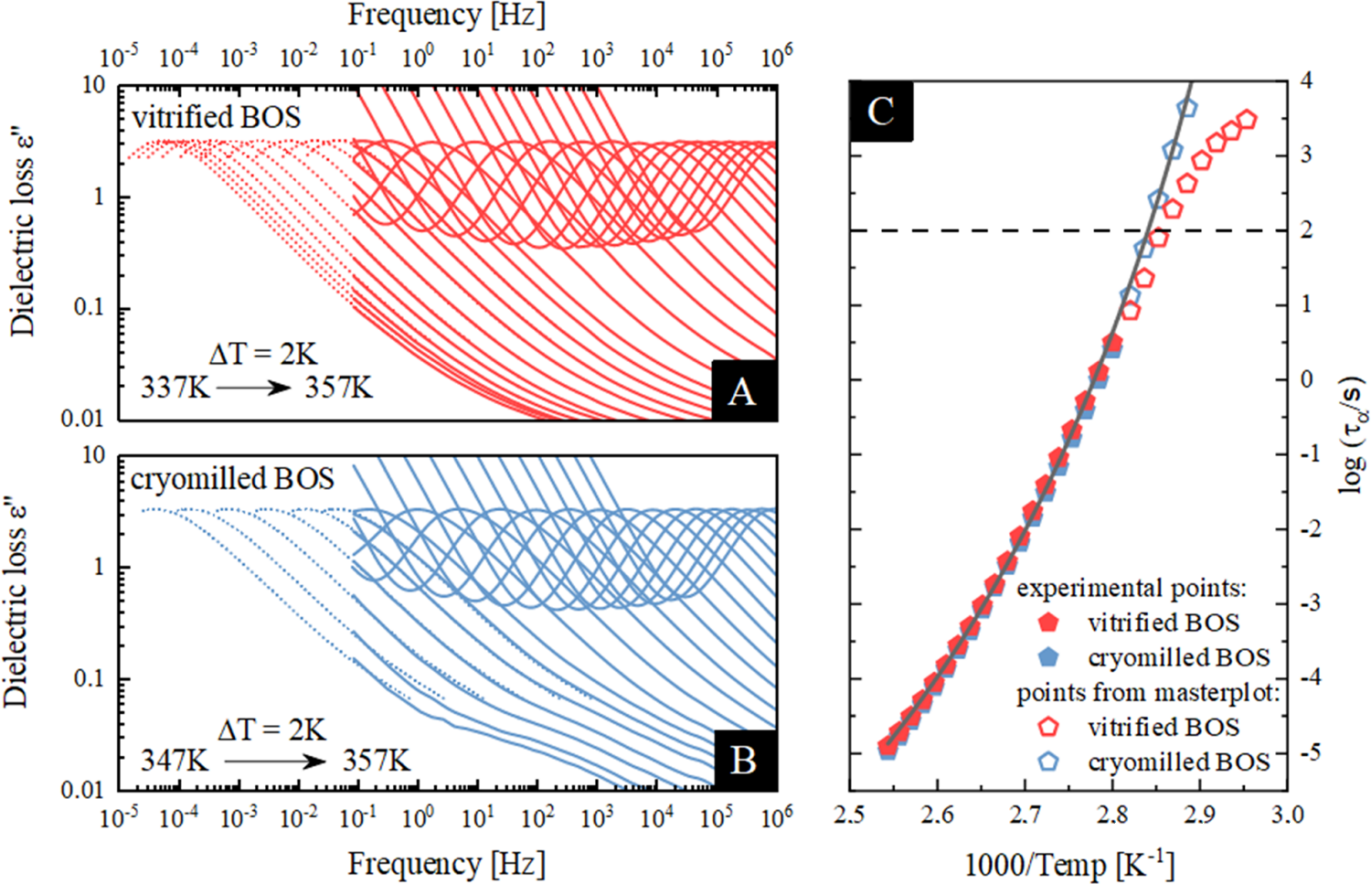
(A, B) Representative dielectric loss spectra of amorphous BOS. Light red lines correspond to the vitrified sample, while light blue lines correspond to the cryomilled one. Dotted lines indicate the so-called master plot method. (C) Relaxation map of BOS. Red and blue pentagons correspond to the vitrified and cryomilled samples. The red and blue empty pentagons represent the τ_α_ values predicted from so-called master plot method for the vitrified and cryomilled API, respectively. The temperature dependence of τ_α_ in the supercooled liquid has been described by the VFT equation and extrapolated to lower temperatures that correspond to the glassy state (gray solid lines).

There is a method allowing the determination of structural relaxation times deeply in the glassy state, which is based on the construction of the so-called master curve.^71^ To obtain such a plot, one has to horizontally shift α-loss peaks obtained at a certain temperature, higher than *T*_g_, to a glassy region, where the α-process is too slow to be experimentally observed (see red and blue dotted lines in Figure 5A,B, respectively). The detailed illustration of this procedure is presented in Figure S6 in the Supporting Information. It should be pointed out that the “master curve” method can be employed only in cases when the shape of the α-process does not change with the increasing temperature, which actually is true in the case of the investigated samples. The performed analysis allowed us to predict the temperature behavior of α-relaxation times in the disordered state of vitrified and cryoground BOS (see Figure 5C).

Interestingly, the obtained results indicated that at *T* < *T*_g_, the cryomilled BOS is characterized by longer relaxation times than in the case of the vitrified one. More importantly, the τ_α_(*T*) of the amorphous sample prepared by cryomilling follows the VFT equation (eq 4) describing the temperature evolution of the structural relaxation above *T*_g_, extrapolated to the amorphous region (see the gray line in Figure 5C). Based on the presented data, it is evident that the cryomilled API shows slower mobility/longer relaxation time below the *T*_g_ (it may suggest higher physical stability) and therefore appears more “aged” or has lower free energy compared to the melt-quenched sample. This was also evident from the extent of enthalpy recovery in DSC thermograms shown in Figure 2a.

**Figure 6:**
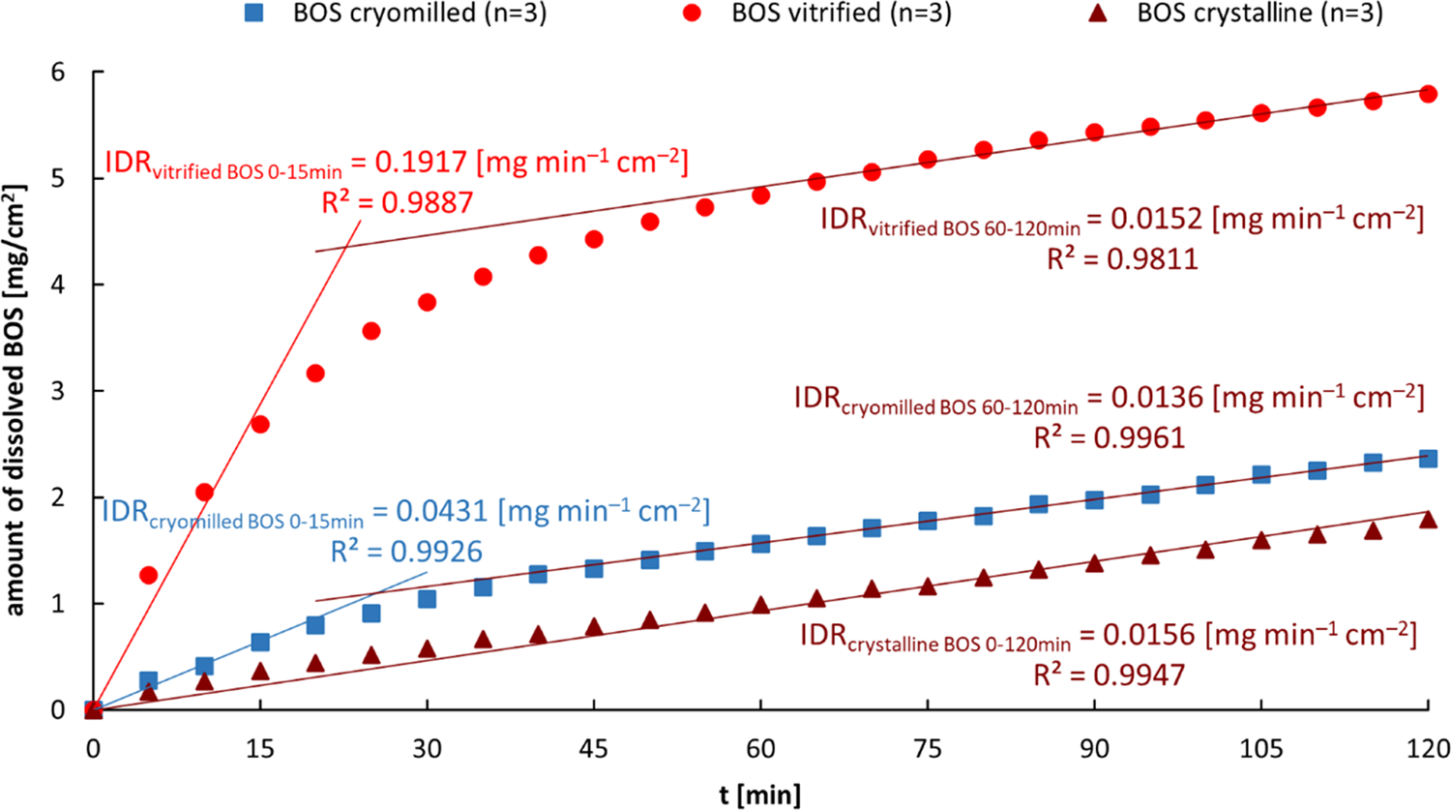
Mean intrinsic dissolution profiles (*n* = 3) of crystalline, vitrified, and cryomilled bosentan (BOS) in 1% w/v aqueous solution of sodium dodecyl sulfate. The intrinsic dissolution rate (IDR) was calculated from each curve’s slope for periods of 0–120 min for crystalline BOS, and 0–15 and 60–120 min for vitrified and cryomilled BOS, respectively (see the text).

As a final point, we decided to check whether there are differences between dissolution rates of both amorphous BOS samples with respect to the crystalline form of the drug. As mentioned in the Introduction section, BOS (a week acid, BCS class IIa drug) is poorly soluble in water but highly permeable through biological membranes. Its bioavailability after oral administration is around 50%.^72^ Increasing the BOS dissolution rate through amorphization can increase the absorption and efficacy of the API. We have performed intrinsic dissolution tests in 1% w/v SDS medium recommended by the Food and Drug Administration (FDA) Dissolution Database. The results of these studies are presented in Figure 6. As expected, the crystalline form of API exhibited a slower dissolution rate from the beginning of the test than its amorphous counterparts. On the other hand, the dissolution behavior of amorphous systems was quite different. Initially, the vitrified BOS dissolution profile showed a 12-fold higher intrinsic dissolution rate of API (IDR = 0.192 ± 0.029 mg min^–1^ cm^–2^) than pure crystalline substance (IDR = 0.016 ± 0.004 mg min^–1^ cm^–2^). On the other hand, cryomilled BOS revealed rather unusual behavior, i.e., it has IDR (0.043 ± 0.008 mg min^–1^ cm^–2^) only 2.7-fold higher than the crystalline one. The higher free energy of amorphous materials is generally associated with increased solubility and dissolution rates. Taking into account the results of thermal and dielectric studies, one can correlate the observed differences in dissolution profiles of both amorphous samples (during the first 15 min of the test) with their physical properties. As shown before, the cryoground BOS is more aged and probably more physically stable compared to the vitrified sample. That might explain its lower IDR (slower dissolution rate), only slightly higher (faster) with respect to the crystalline form of API.

Interestingly, after 40–60 min of the test, both amorphous materials changed their dissolution kinetics significantly, and their dissolution rate after 60 min was similar to that for crystalline substance. Such behavior in an aqueous environment, and almost identical IDR values as the crystalline form, may indicate the conversion of amorphous forms back to the crystalline monohydrate. Importantly, such a solvent-mediated phase transformation of anhydrous APIs has also been reported by other authors^73−78^ (BOS molecule has a high value of a solvent-accessible surface area,^55^ and this property may cause a quick phase conversion). However, it should be noted that the extent of dissolution is still considerably higher in the melt-quenched sample and is not drastically decreased. This does indicate that the rate of conversion to the crystalline form in the vitrified sample is slower compared to the dissolution of the amorphous API in the medium.

## Conclusions

In the present study, amorphous BOS obtained by two methods, vitrification and cryomilling, was thoroughly characterized. X-ray diffraction and infrared spectroscopy examinations showed that both disordered samples are very similar in terms of the local structure and H-bonding scheme. Dielectric studies revealed that they are also characterized by the same shape of the structural (α)-peak as well as molecular dynamics at *T* > *T*_g_. Moreover, the analysis of temperature dependence of α-relaxation times (predicted for both samples at *T* < *T*_g_ from the master curve analysis), like the results of calorimetric measurements, indicated that cryoground BOS is more aged and probably more physically stable compared to the vitrified API. Importantly, the differences in physical properties of both amorphous systems influenced their dissolution rates in 1% w/v aqueous solution of sodium dodecyl sulfate (IDR of the quenched sample at first 15 min of the test was clearly higher with respect to the cryomilled one). Furthermore, FTIR data, together with the results of the TGA method clearly showed that cryomilling leads to the formation of nearly anhydrous amorphous BOS. This finding is different from that reported by Megarry et al.^56^ (the authors obtained hydrated disordered TRE during the mechanical treatment of this saccharide). To explain the discrepancy between our results and those presented in the paper,^56^ we considered the difference in the chemical structure of TRE (hydrophilic system) and BOS (hydrophobic system), as well as the type of vessel used for milling of the sample. We hypothesize that in the hermetically closed vessel, that is cooled from *T* = 298 to 80 K, there is a strong decrease in pressure. This fact together with the release of the water from the crystalline lattice of BOS hydrate generates conditions mimicking the ones applied during freeze-drying. Therefore, water removal proceeds via the sublimation of ice at very low temperatures and pressures. In the mills, where the vessel is not hermetically closed, such an effect is not detected. We think that the results presented herein will initiate an interesting discussion on the behavior of milled/cryomilled drug substances and the mechanism of amorphization behind this experimental technique.

## Supporting Information Available

XRD data (diffractograms measured in the wide 2θ range for the crystalline, vitrified and cryoground BOS as well as comparison of the structure factors and atomic pair distribution functions for amorphous samples), results of FTIR measurements and DFT calculations for the examined systems, comparison of the normalized dielectric loss spectra collected at different temperatures above *T*_g_ for the vitrified and cryomilled BOS, as well as the presentation of the master plot method with respect to the data obtained for these systems (PDF)
