# Supplementary material for: Studies on the Vitrified and Cryomilled Bosentan
Source: Mol Pharm. 2021 Dec 1;19(1):80–90. doi: 10.1021/acs.molpharmaceut.1c00613 (PMC8728735; doi:10.1021/acs.molpharmaceut.1c00613)
Supplement: Supplementary file 1 — mp1c00613_si_001.pdf [file mp1c00613_si_001.pdf]

Studies on the Vitrified and Cryomilled Bosentan

Aldona Minecka[a,*], Krzysztof Chmiel[a], Karolina Jurkiewicz[b], Barbara Hachuła[c], Rafał Łunio[d], Daniel Żakowiecki[e], Kinga Hyla[f], Bartłomiej Milanowski[f,g], Kajetan Koperwas[b], Kamil Kamiński[b], Marian Paluch[b], Ewa Kamińska[a,*]

[a] *Department of Pharmacognosy and Phytochemistry, Faculty of Pharmaceutical Sciences in Sosnowiec, Medical University of Silesia in Katowice, 41-200 Sosnowiec, Poland*

[b] *Institute of Physics, Faculty of Science and Technology, University of Silesia in Katowice, 41-500 Chorzów, Poland*

[c] *Institute of Chemistry, University of Silesia in Katowice, 40-006 Katowice, Poland*

[d] *Polpharma SA, 83-200 Starogard Gdański, Poland*

[e] *Chemische Fabrik Budenheim KG, Rheinstrasse 27, 55257, Budenheim, Germany*

[f] *Chair and Department of Pharmaceutical Technology, Faculty of Pharmacy, Poznan University of Medical Sciences, 60-780 Poznan, Poland*

[g] *GENERICA Pharmaceutical Lab, 64-360 Zbąszyń, Poland*

Supporting Information

XRD data

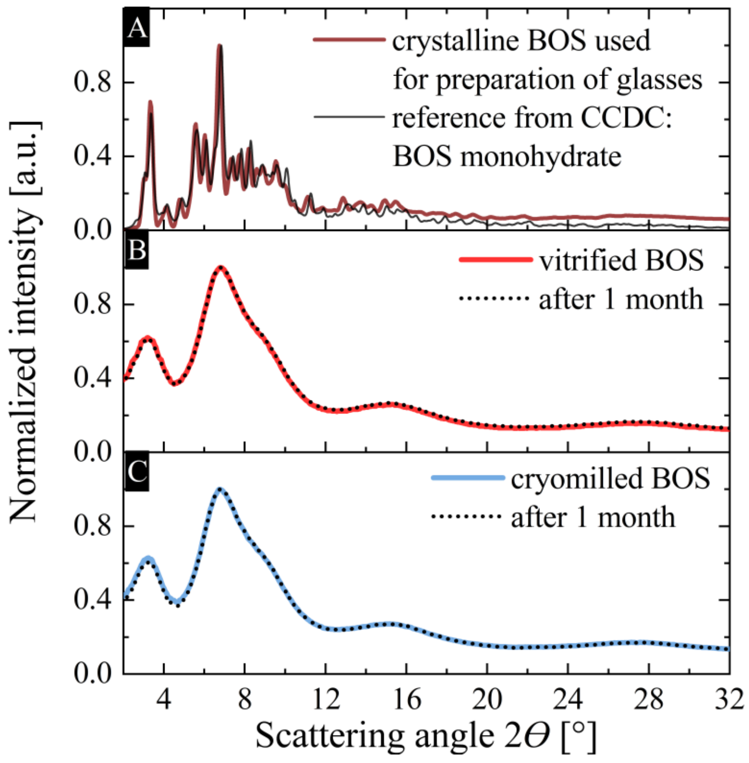

Figure S1. Comparison of X-ray diffraction patterns measured for (A) crystalline BOS monohydrate used to prepare vitrified and cryomilled samples and its reference from CCDC database (no. 920210), (B) vitrified BOS just after preparation and after 1 month of storage, (C) cryomilled BOS just after preparation and after 1 month of storage.

The collected X-ray diffraction patterns (Figure S1) were converted to the functions of the scattering vector, $Q = 4\pi(sin\theta)/\lambda$, where 2θ is the scattering angle and the wavelength of the incident beam, λ, is equal to 0.56 Å. The intensity functions were corrected for background, polarization, absorption, incoherent Compton scattering and normalized to the electron units. Then, the diffraction data were transformed to the structure factor $S(Q) = \frac{I(Q)-(<f^2>-<f>^2)}{<f>^2}$, where: $I(Q)$ is the coherently scattered intensity normalized to electron units, $<f^2>= \sum_{i=1}^{n} c_i f_i^2$, $<f>= \sum_{i=1}^{n} c_i f_i$, c_i and f_i are the concentration and the atomic scattering factor of the i-th atomic species, respectively, and n is the number of atomic species in the sample. Finally, the diffraction data in reciprocal space were converted to a real space representation in the form of the atomic pair distribution function $PDF(r) = \frac{2}{\pi}\int_0^{Q_{max}} Q[S(Q)-1]W(Q)\sin(Qr)\,dQ$, where Q_{max} indicates the maximum value of Q achieved in the experiment (here Q_{max} = 20 Å^{-1}) and $W(Q) = \sin(\pi Q/Q_{max})/(\pi Q/Q_{max})$ is the function used to minimize truncation oscillations.

The comparison of the $S(Q)$ and $PDF(r)$ for vitrified and cryomilled BOS presented in Figure S2 shows that their local structure is very similar.

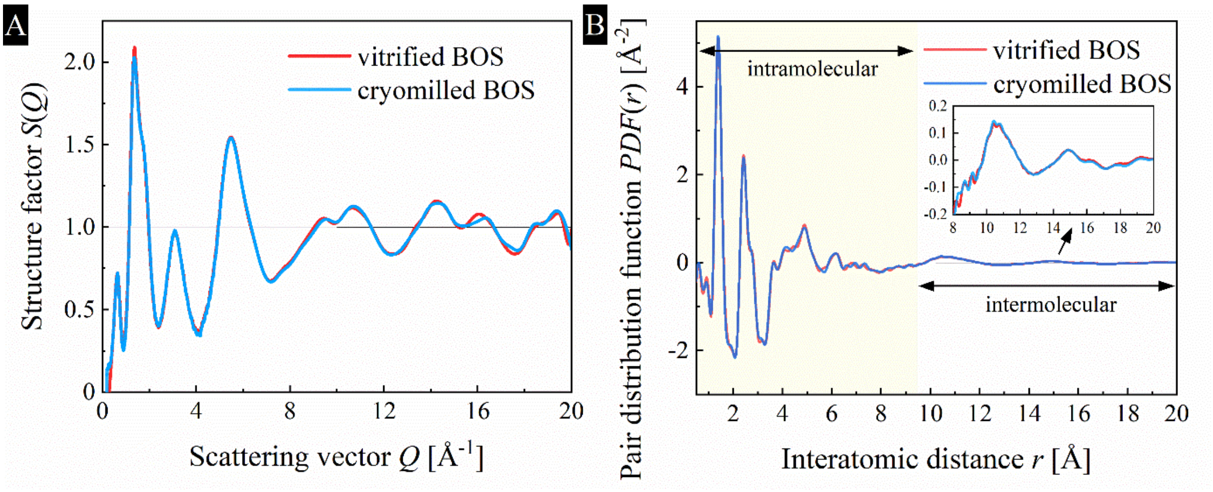

Figure S2. Comparison of the structure factors (A) and atomic pair distribution functions (B) for amorphous BOS samples produced using vitrification and cryomilling methods.

FTIR data

According to the literature data, the supramolecular structure of BOS·H_2O is stabilized by O—H···O, O—H···N and N—H···O hydrogen bonds (HBs) with a single water (W) molecule and weak O—H···N intermolecular interactions between the hydroxyl group and one of the nearby pyrimidine rings (Figure S3).[1,2] It should be noted that the HBs involving water molecules are shorter (N-H···O(W) 2.721 Å; O(W)-H(W)···N 2.873 Å) than those existing between the BOS molecules (O-H···N 3.317 Å; O-H···N 3.141 Å). Thus, it is expected that the band originating from the stretching vibrations of X—H (X=O, N) groups, characterized by a greater length (shorter HBs), should be shifted toward lower frequencies compared to the band corresponding to the H-bonded X-H groups occurring between the BOS molecules.

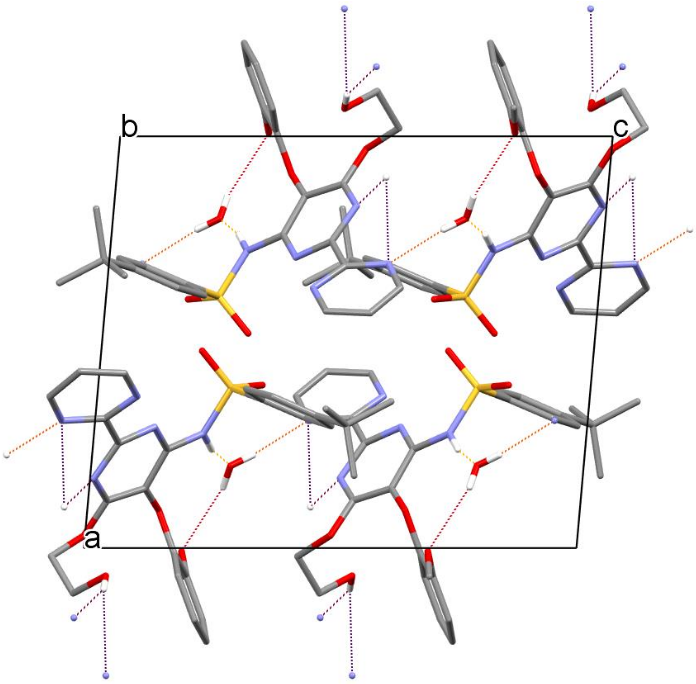

Figure S3. Part of the crystal structure of BOS·H_2O, showing the formation of two-dimensional supramolecular assemblies in the *ac* plane. All H atoms not involved in hydrogen bonding have been removed for clarity.

As shown in panels A and B of Figure 3 (main manuscript), the high-frequency spectrum of the crystalline BOS·H_2O (wine line) exhibits a broad band between 3700-2400 cm^{-1} assigned to the stretching vibrations of H-bonded X–H groups of BOS·H_2O molecules ($\nu_{X\text{-}H}$). Based on the crystallographic data,[1,2] the higher energetic component of the X-H band (above 3000 cm^{-1}) can be assigned to the vibrations of X-H groups of weaker H-bonds (occurring between BOS molecules), while an intense shoulder occurring ca. 3000-2400 cm^{-1} can be related to the stretching vibrations of N-H groups involved in the formation of stronger

H-bonds between BOS and water molecules. A similar O-H band component at lower frequency was also detected in the FTIR spectrum of 3,4-dimethoxyphenyl)acetic acid monohydrate, and it was absent for the anhydrous form of this compound.[3] Moreover, the wide $\nu_{X\text{-}H}$ band is disturbed by the intense peaks originating from_the C–H stretching vibrations of the aromatic ring (3013, 3046, 3066, and 3093 cm^{-1}) and the C–H stretching vibrations of aliphatic C-H groups (2962, 2906, 2870, 2837 cm^{-1}). Importantly, in this spectrum, the characteristic peak occurring at 3626 cm^{-1} is observed. Generally, the non-associated ('free') hydroxyl groups absorb strongly in the frequency region of 3670-3580 cm^{-1}.[4] However, in the case of the hydrates, the presence of $\nu_{X\text{-}H}$ band at a higher frequency (above 3600 cm^{-1}) can be attributed to the interactions between interlayer H_2O and the compound.[5,6,7,8,9] Thus, in BOS monohydrate, the water molecules can be presented in the interlayer space, tending to associate with BOS molecules and to complete the filling of the interlayer space. In turn, the characteristic absorption peaks occurring at 1169 cm^{-1} (symmetric stretching vibrations of SO_2 group), 1341 cm^{-1} (asymmetric stretching vibrations of SO_2 group), 1251 and 1020 cm^{-1} (the vibrations of aromatic and aromatic-aliphatic ether groups) as well as 1579, 1558, 1504 and 1442 cm^{-1} (the vibrations from pyrimidine rings) are detected in the lower frequency range.[2]

DFT calculations

To verify the band assignment for the FTIR spectrum of the crystalline BOS monohydrate, especially in the higher frequency region, Density Functional Theory (DFT) calculations were carried out for the part of the crystalline structure of the studied compound, see Figure S4. It should be mentioned that the calculations of the optimized geometry of BOS monohydrate were performed by the Gaussian09 package using the standard B3LYP/6-31G(d,p) model[10] starting from the experimental structure of this compound.[11] To generate intermolecular interactions between a water molecule and surrounding neighbors (two BOS molecules), the geometry was optimized for a molecular structure consisting of one water molecule, one BOS molecule, and the fragment of the second BOS molecule involving the pyrimidine ring.

A comparison of the experimental (red line) and theoretical (blue line) IR spectra, presented in Figure S4 B, shows a fairly good agreement. As expected, our computations indicated that the peak at 3629 cm^{-1} is assigned to H-bonded water molecules, while the absorption signal at 2831 cm^{-1} corresponds to H-bonded N-H groups of BOS. On the other hand, the OH group of BOS molecule does not participate in the H-bonding with a water

molecule, thus it was treated as "free" during the calculations. As a result, the $\nu_{OH\ free}$ band from the BOS molecule was also observed above 3600 cm^{-1} in the theoretical spectrum. In fact, in the crystalline BOS monohydrate, this absorption signal is red-shifted as weak O—H···N intermolecular interactions between the hydroxyl group and one of the pyrimidine rings exist.

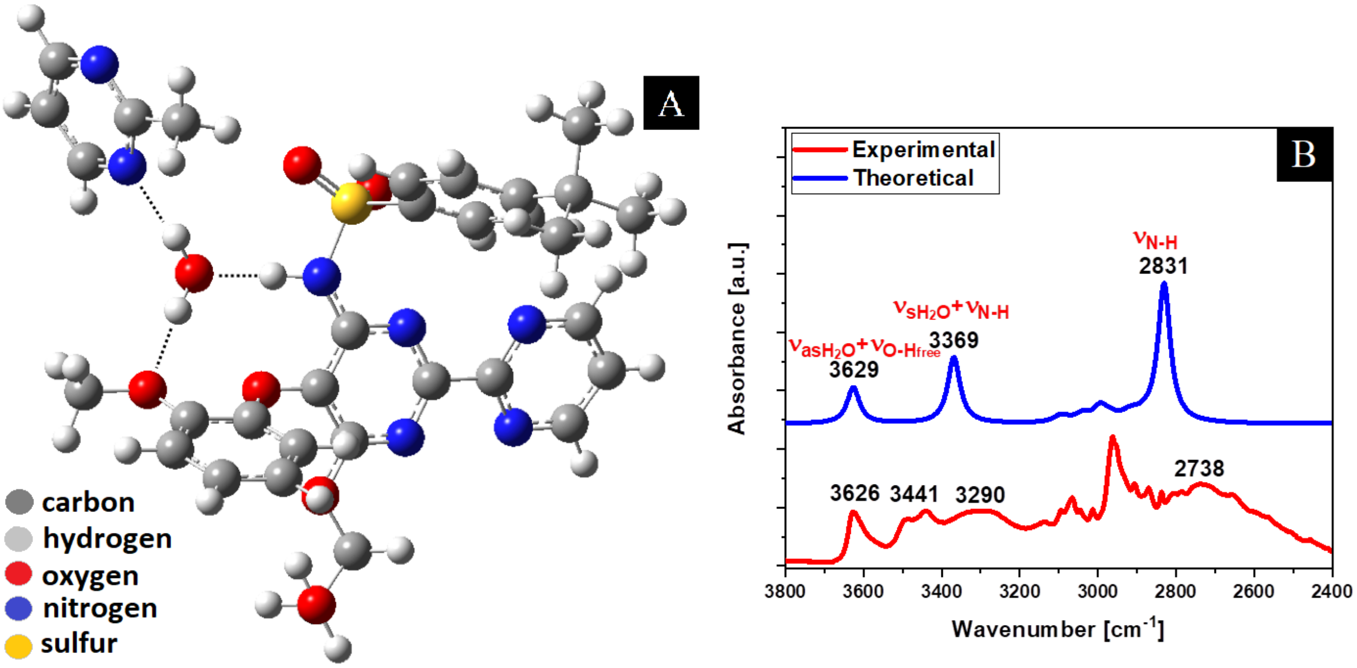

Figure S4. Panel (A) shows the part of the crystalline structure of BOS monohydrate optimized by the density functional theory (DFT). Panel (B) presents experimental (red) and theoretical (blue) FTIR spectra of BOS monohydrate in the 3800-2400 cm^{-1} frequency range.

BDS data

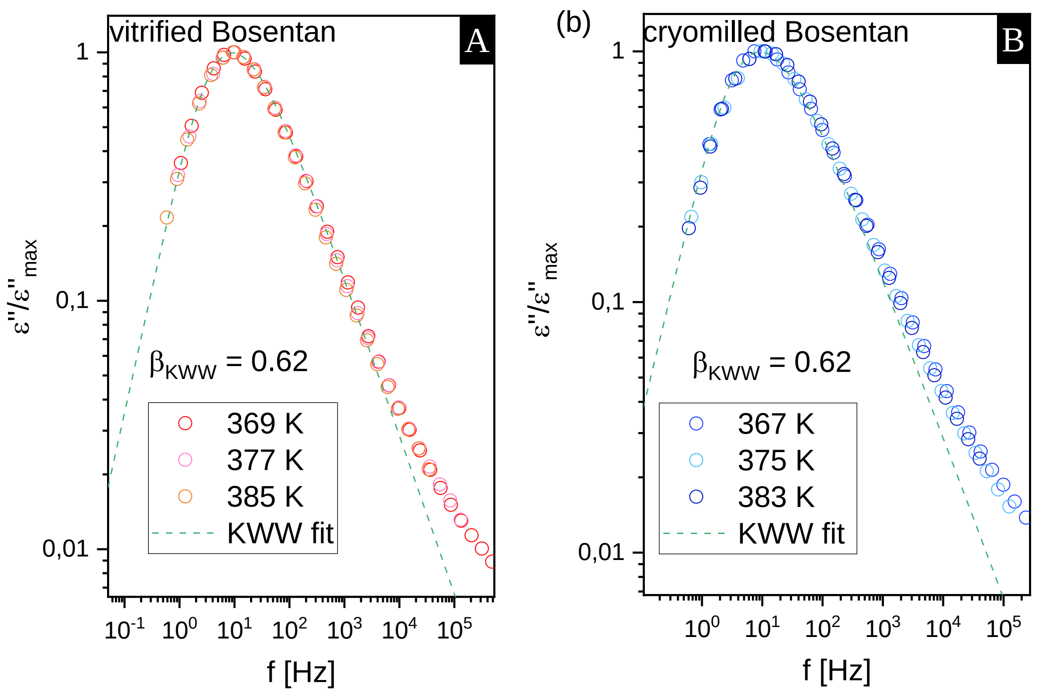

Figure S5. A comparison of the normalized dielectric loss spectra collected at different temperatures above T_g for both vitrified (A), and cryomilled (B) BOS. The dc-conductivity part was subtracted from the measured loss spectra. Dotted lines represent KWW fits.

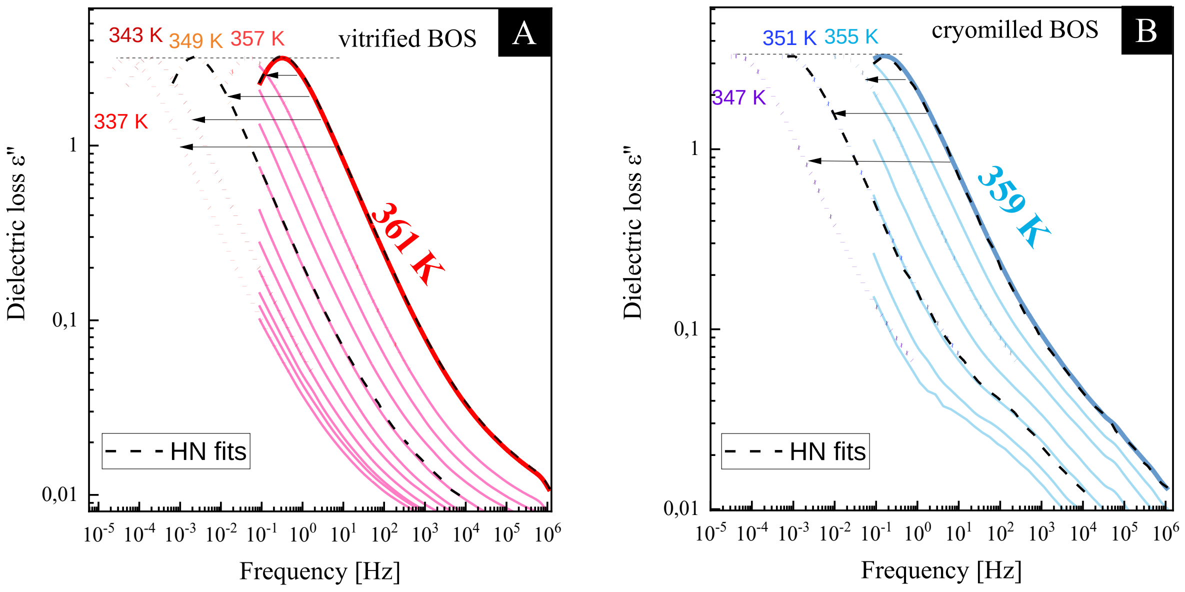

Figure S6. The presentation of the *master plot* method. (A): The spectrum measured for vitrified BOS at 361 K was shifted horizontally to superpose with those collected at lower T: 357 K, 349 K, 343 K and 337 K, where the α-loss peak was not observed in the experimental window (B): The spectrum measured for cryomilled BOS at 359 K was shifted horizontally to superpose with those obtained at 355 K, 351 K, and 347 K. Analysis of these new spectra using the HN function (black dotted lines) enabled us to determine structural relaxation times at T close and below the T_g for both samples (open pentagons in Figure 5C in the main text of the manuscript).

(1) Kaur, M.; Jasinski, J. P.; Keeley, A. C.; Yathirajan, H. S.; Betz, R.; Gerber, T.; Butcher, R. J. Bosentan monohydrate. *Acta Crystallogr. Sect. E Struct. Reports Online.* **2013**, *69,* o12–o13. doi:10.1107/S1600536812048969.

(2) Krupa, A.; Majda, D.; Mozgawa, W.; Szlęk, J.; Jachowicz, R. Physicochemical properties of bosentan and selected PDE-5 inhibitors in the design of drugs for rare diseases. *AAPS PharmSciTech.* **2017**, *18,* 1318–1331. doi:10.1208/s12249-016-0599-7.

(3) Hachuła, B.; Nowak, M.; Kusz, J. Hydrogen-bonding interactions in (3,4-dimethoxyphenyl)acetic acid monohydrate. *Acta Crystallogr. Sect. C Cryst. Struct. Commun.* **2008**, *64,* o357–o360. doi:10.1107/S0108270108014157.

(4) Socrates, G. *Infrared characteristic group frequencies: Tables and charts, 3rd edition..* John Wiley and Sons: Ltd, Chichester, 2001. ISBN: 978-0-470-09307-8.

(5) Byrn, S. R.; Zografi, G.; Chen, X. (S). *Solid-state properties of pharmaceutical materials.* John Wiley & Sons: 2017, pp. 40. ISBN: 978-1-118-14530-2 http://www.wiley.com/en-pl/Solid+State+Properties+of+Pharmaceutical+Materials-p-9781118145302.

(6) Parry, S. A.; Pawley, A. R.; Jones, R. L.; Clark, S. M. An infrared spectroscopic study of the OH stretching frequencies of talc and 10-Å phase to 10 GPa. *Am. Min.* **2007**, *92,* 525–531. doi:10.2138/am.2007.2211.

(7) Falk, M.; Huang, Ch. -H. Infrared spectra of water in crystalline hydrates: $KSnCl_3 \cdot H_2O$, an untypical monohydrate. *Can. J. Chem.* **1974**, *52,* 2928–2931. doi:10.1139/v74-427.

(8) Madejová, J. FTIR techniques in clay mineral studies. *Vib. Spectrosc.* **2003**, *31,* 1–10. doi:10.1016/S0924-2031(02)00065-6.

(9) Farmer, V. C. (Ed.) *The infrared spectra of minerals.* Mineralogical Society: London, UK, 1974, p. 331. doi:10.1180/mono-4.

(10) Frisch, M. J.; Trucks, G. W.; Schlegel, H. B.; Scuseria, G. E.; Robb, M. A.; Cheeseman, J. R.; Scalmani, G.; Barone, V.; Mennucci, B.; Petersson, G. A.; Nakatsuji, H.; Caricato, M.; Li, X.; Hratchian, H. P.; Izmaylov, A. F.; Bloino, J.; Zheng, G.; Sonnenberg, J. L.; Hada, M.; Ehara, M.; Toyota, K.; Fukuda, R.; Hasegawa, J.; Ishida, M.; Nakajima, T.; Honda, Y.; Kitao, O.; Nakai, H.; Vreven, T.; Montgomery, J. A. Jr.; Peralta, J. E.; Ogliaro, F.; Bearpark, M.; Heyd, J. J.; Brothers, E.; Kudin, K. N.; Staroverov, V. N.; Kobayashi, R.; Normand, J.; Raghavachari, A.; Rendell, J. C.; Burant, S. S.; Iyengar, J.; Tomasi, M.; Cossi, N.; Rega, J. M.; Millam, K.; Klene, M.; Knox, J. E.; Cross, J. B.; Bakken, V.; Adamo, C.; Jaramillo, J.; Gomperts, R.; Stratmann, O.; Yazyev, A. J.; Austin, R.; Cammi, C.; Pomelli, J. W.; Ochterski, R. L.; Martin, K.; Morokuma, R. E.; Zakrzewski, V. G.; Voth, G. A.; Salvador, P.; Dannenberg, J. J.; Dapprich, S.; Daniels, A. D.; Farkas, Ö.; Foresman, J. B.; Ortiz, J. V.; Cioslowski, J.; Fox, D. J. Gaussian 09, Gaussian, Inc., Wallingford CT, 2009.

(11) Kaur, M.; Jasinski, J. P.; Keeley, A. C.; Yathirajan, H. S.; Betz, R.; Gerber, T.; Butcher, R. J. Bosentan monohydrate. *Acta Crystallogr. Sect. E Struct. Reports Online.* **2013**, *69,* o12–o13. doi:10.1107/S1600536812048969.
